# Etude séro-epidémiologique de trois infections sexuellement transmissibles (Chlamydia Trachomatis, Hépatite B, Syphilis): cas de l’Hôpital de District de Nkoldongo à Yaoundé

**DOI:** 10.11604/pamj.2016.25.244.11107

**Published:** 2016-12-20

**Authors:** Marie Chantal Ngonde Essome, Bonglaisin Julius Nsawir, Rodrigue Dongang Nana, Patrick Molu, Mansour Mohamadou

**Affiliations:** 1 Institut de Recherches Médicales et d’Etudes de Plantes Médicinales, Ministère de la Recherche Scientifique et de l’Innovation, Yaoundé, Cameroun; 2 Centre hospitalo Universitaire, Yaoundé, Cameroun

**Keywords:** Chlamydia trachomatis, hépatite virale B, Syphilis, femmes, Cameroun, Chlamydia trachomatis, Viral hepatitis B, syphilis, women, Cameroon

## Abstract

**Introduction:**

Les infections sexuellement transmissibles sévissent toujours dans les pays en voie de développement et particulièrement au Cameroun. Le but de notre étude est de déterminer la distribution des infections sexuellement transmissibles suivantes: l’hépatite virale B, le *Chlamydia trachomatis* et de la syphilis dans une population de femmes venant consulter spontanément à l’Hôpital de District de Nkoldongo à Yaoundé, d’évaluer d’éventuelles coïnfections entre ces trois affections et de ressortir les connaissances de ces femmes sur leur mode de transmission sexuelle.

**Méthodes:**

Notre étude prospective et descriptive a porté sur 182 femmes dont l’âge variait entre 18 et 48 ans. Les femmes ont été testées sérologiquement pour le *Chlamydia trachomatis* par une méthode ELISA (kit des laboratoires General Biological Corp). L’hépatite virale B a été dépistée par une méthode immunochromatographique (kit des laboratoires Human) et la syphilis par une méthode d’agglutination en ce qui concerne le RPR (Kit des laboratoires Biocentric) et le TPHA (kit des laboratoires Human).

**Résultats:**

Nos résultats ont montré que: la distribution du *Chlamydia trachomatis*, de l’hépatite virale B et la syphilis a été respectivement de 22,52%, 4,39%, 0,54%.De plus, nous avons observé une coinfection *Chlamydia trachomatis* hépatite virale B avec un taux de 2,74%. Par ailleurs la réinfection au *Chlamydia trachomatis* a été rencontrée dans 4,94% de cas. S’agissant du mode de transmission de ces affections 67,57% et 70,87% de femmes ne connaissaient pas la voie de transmission sexuelle pour le *Chlamydia trachomatis* et pour l’hépatite virale B respectivement, tandis que 91,2 % des femmes connaissaient la voie de transmission sexuelle pour la syphilis.

**Conclusion:**

Le diagnostic d’une infection à *Chlamydia trachomatis* chez une patiente doit susciter le dépistage de l’hépatite virale B.

**Introduction:**

Sexually transmitted infections are still frequent in developing countries and particularly in Cameroon. The aim of this study was to determine the distribution of the following sexually transmitted infections: viral hepatitis B, Chlamydia trachomatis and syphilis in a population of women spontaneously visiting the Nkoldongo District Hospital in Yaoundé as well as to evaluate possible co-infections among these three conditions and to bring out women’s prior knowledge of how sexual transmission occurs.

**Methods:**

We conducted a prospective and descriptive study including 182 women aged between 18 and 48 years. These women underwent serologic testing for Chlamydia trachomatis with ELISA (General Biological Corp laboratory test kit. Hepatitis B virus was detected using immunochromatographic method (Human laboratory kit) while syphilis was detected using RPR agglutination (Biocentric Laboratories kit )and TPHA agglutination (Human laboratory kit) method.

**Results:**

Our results showed that the distribution of Chlamydia trachomatis, viral hepatitis B and syphilis was 22.52%, 4.39%, 0.54% respectively. Moreover, we reported a Chlamydia trachomatis and Viral hepatitis B coinfection rate of 2.74%. In addition, Chlamydia trachomatis reinfection was detected in 4.94% of cases. Regarding the mode of transmission of these infections, 67.57% and 70.87% of women didn’t know how Chlamydia trachomatis and viral hepatitis sexual transmission could occur respectively, while 91.2% of women knew how was syphilis spread.

**Conclusion:**

The diagnosis of chlamydia trachomatis infection should prompt screening for viral hepatitis B.

## Introduction

L’épidémiologie des infections sexuellement transmissibles n’est pas bien connue au Cameroun. L’hépatite virale B, dont un des modes de transmission est sexuelle, constitue un problème important de santé publique dans certaines régions du monde [1]. Les complications d’une hépatite chronique sont la cirrhose du foie et le carcinome hépatocellulaire. L’Afrique subsaharienne et l’Asie de l’Est demeurent les régions du monde les plus touchées par cette infection virale, on y trouve entre 5-10% d’adultes chroniquement infectés [1]. Le Cameroun est aussi une zone de haute endémicité pour l’hépatite virale B. Une étude menée par Bigna et al [2] a trouvé une prévalence de 10,2% parmi les femmes enceintes dans un milieu rural au Cameroun. Une autre infection bactérienne également transmissible sexuellement, le *Chlamydia trachomatis* sévit avec une prévalence élevée au Cameroun. Elle est responsable de salpingite, de cervicite, de maladies inflammatoires pelviennes et d’infertilité. Des études menées par Sende et al. [3] ont rapporté des prévalences respectives de 8,6% et de 11,4% parmi les femmes enceintes et les femmes infertiles camerounaises. Enfin la syphilis connait une recrudescence indiscutable de part le monde. On estime à près de 4 millions le nombre de cas de syphilis en Afrique sub saharienne et à près de 6 millions en Asie du Sud Est [4]. Une étude menée par Taiwo et al. [5] au Nigéria a rapporté une prévalence de la syphilis de 2,97 % chez les femmes enceintes, tandis que Bouba et al. [6] ont retrouvé une prévalence de 5,5% chez les femmes du Nord-Cameroun Notre étude a pour but de déterminer la distribution des infections sexuellement transmissibles suivantes: l’hépatite virale B, le *Chlamydia trachomatis* et de la syphilis dans une population de femmes venant consulter spontanément à l’Hôpital de District de Nkoldongo situé en zone urbaine de Yaoundé, d’évaluer d’éventuelles coïnfections entre ces trois affections et de ressortir les connaissances de ces femmes sur leur mode de transmission sexuelle.

## Méthodes

**Matériels:** Notre étude prospective et descriptive a été conduite à l’Hôpital de District de Nkoldongo de Yaoundé de Février à Novembre 2015.L’approbation du comité d’éthique a été obtenue. Notre échantillon était composé de 182 femmes âgées de 18 à 48 ans. Les critères d’inclusion étaient les suivants : toute patiente consentante, sexuellement active depuis au moins un an et venant consulter spontanément à l’Hôpital de District de Nkoldongo. Etaient exclues de l’étude toute patiente sous antibiothérapie depuis moins de 4 semaines et/ou qui refuse de participer volontairement à l’étude. Une fiche technique de renseignements nous a permis de collecter les données sociodémographiques des patientes (âge, niveau d’instruction), les antécédents médicaux (infection sexuellement transmissible antérieure, traitement reçu), la connaissance sur le mode de transmission sexuelle de ces trois infections. Une fois incluse dans l’étude, un prélèvement de 5 ml de sang veineux sur tube sec a été effectué sur tous les sujets.

**Méthodes:** La détection de l’antigène HBS (AgHBs) sur chaque sérum a été faite par la méthode immunochromatographique en utilisant le kit HEXAGON HBsAg des laboratoires HUMAN (Max-Planck-Ring 21-D-65205 Wiesbaden-Germany).Tout résultat considéré positif (Ag HBs>1 U/ml) montrait deux lignes colorées dans la zone réactionnelle, tandis qu’un résultat négatif montrait uniquement la ligne témoin. Le dosage des anticorps IgG et IgM anti-*Chlamydia trachomatis* sur chaque sérum a été réalisé en utilisant la méthode ELISA en utilisant le kit des laboratoires General Biologicals Corp. (#6, Innovation First Road, Scienced Based Industrial Park, HSIN CHU 30077, Taiwan, R.O.C). Le test était considéré positif pour un index supérieur à 1.Toutefois dans notre étude nous avons retenu un index en anticorps supérieur 2 afin d’éviter les cicatrices sérologique. De plus le test était considéré négatif pour un index inférieur à 0,9.Toute patiente ayant un index compris entre 0,9 et 2 a subi un autre test deux semaines plus tard. Nous avons effectué un test d’agglutination, le RPR (test rapide de détection des réagines dans le sérum ou le plasma) pour dépister la syphilis en utilisant le kit fourni par les laboratoires Biocentric (Zone d’Entreprise du Val d’Aran 83150 Bandol-France), puis un test d’hémagglutination pour confirmation, le TPHA (le *Treponema Pallidum* Haemagglutination Test) en utilisant le kit des laboratoires HUMAN (MAX Planck-Ring 21-D-65205 Wiesbaden-Germany. Le seuil positif retenu pour le RPR a été de 1/16 et de 1/1280 pour le TPHA afin d’éliminer les sérologies cicatricielles. Les informations recueillies ont été étudiées à l’aide du logiciel Epi-info version 3.5.4. Pour les différentes variables, nous avons fait une analyse descriptive.

## Résultats

Nous avons inclus dans notre étude 182 femmes dont l’âge moyen était de 33,76 ans. Il ressort du Tableau 1 que la distribution de l’hépatite virale B était de 4,39%, soit 8/182 femmes. Par ailleurs 41 femmes ont été testées positives au *Chlamydia trachomatis*, soit une une distribution de 22,52%, et nous avons observé 9 réinfections soit un taux de 4,94%. En outre une seule femme a été testée positive à la fois au RPR et au TPHA, soit une distribution de 0,54% pour la syphilis. De plus 5/182 femmes (2,74%) ont été testés positives concomitamment pour l’infection à *Chlamydia trachomatis* et pour l’infection à hépatite virale B (Tableau 1). L’analyse des données socio démographiques a montré que 28,02% de femmes étaient analphabètes tandis que 71,97% d’entre elles avaient au moins le niveau primaire et étaient reparties comme suit : 37,36 % avaient le niveau primaire, 23,07% avaient le niveau secondaire et 11,53% avaient fait des études universitaires (Tableau 2). S’agissant du mode de transmission de ces affections 67,57% et 70,87% de femmes ne connaissaient pas la voie de transmission sexuelle pour le *Chlamydia trachomatis* et pour l’hépatite virale B respectivement, tandis que 91,2 % des femmes connaissaient la transmission sexuelle pour la syphilis.69,23% de femmes éduquées connaissaient la transmission sexuelle de la syphilis contre 25,27% et 30,21% pour l’hépatite B et le *Chlamydia trachomatis* respectivement (Tableau 2).

**Tableau 1:** Répartition des femmes selon l’âge et les différentes infections

	CHL		HB		S		CHL/HB	
AGE (ans)	I (%)	NI(%)	I(%)	NI(%)	I(%)	NI(%)	I(%)	NI(%)
[18-23[	11(6,04)	29(15,93)	3(1,64)	37(20,32)	-	40(21,97)	2(1,09)	38(20,87)
[23-28[	9(4,94)	30(16,48)	1(0,54)	38(20,87)	-	39(21,42)	-	39(21,42)
[28-33[	5(2,74)	29(15,93)	1(0,54)	33(18,13)	-	34(18,68)	1(0,54)	33(18,13)
[33-38[	4(2,19)	18(9,89)	-	22(12,08)	1(0,54)	21(11,53)	-	22(12,08)
[38-43[	5(2,74)	12(6,59)	2(1,09)	15(8,24)	-	17(9,34)	2(1,09)	15(8,24)
[43-48]	7(3,84)	23(12,63)	1(0,54)	29(15,93)	-	30(16,48)	-	30(16,48)
Sous total (%)	41(22,52)	141(77,47)	8(4,39)	174(95,60)	1(0,54)	181(99,45)	5(2,74)	177(97,25)
Total (%)	182(100)		182(100)		182(100)		182(100)	

CHL:Chlamydia trachomatis HB:Hépatite virale B S: Syphilis I: Infectées

NI:Non infectées

**Tableau 2:** Connaissance des femmes sur le mode de transmission sexuelle du Chlamydia Trachomatis, la syphilis et l’hépatite B

Infection	CHL				HB				S			
Niveau d’éducation	AN		ED		AN		ED		AN		ED	
Nombre de femmes connaissant le mode de transmission sexuelle (%)	oui	non	oui	non	oui	non	oui	non	oui	non	oui	non
	4(2,19)	47(24,82)	55(30,21)	76(41,75)	7(3,84)	44(24,17)	46(25,27)	85(46,70)	40(21,97)	11(6,04)	126(69,23)	5(2,74)
Sous total (%)	51(28,02)		131(71,97)		51(28,02)		131(71,97)		51(28,02)		131(71,97)	
Total (%)	182 (100)				182 (100)				182 (100)			

AN : Analphabètes ; ED : Eduquées ; CHL : Chlamydia ; HB : Hépatite virale B ; S : Syphilis

## Discussion

Les infections sexuellement transmissibles sont toujours d’actualité dans le monde et plus particulièrement en Afrique. En effet l’hépatite virale B constitue un important problème de santé publique dans la plupart des pays en voie de développement [7]. Le Cameroun n’en est pas épargné. Bien qu’une grande partie de cette infection se transmet par voie sanguine et notamment périnatale [1], la transmission sexuelle de l’hépatite virale B n’est pas négligeable dans les pays de forte endémicité. Dans notre étude la distribution de l’hépatite B a été de 4,39% chez nos femmes venues se faire consulter spontanément à l’hôpital de district de Nkoldongo. Notre résultat est moins élevé que celui de Bchir et al [8] qui trouvent une prévalence de 6,1% dans un groupe de prostituées en Tunisie. Ceci pourrait s’expliquer par le fait que ces femmes (les prostituées) constituent un groupe à risque et ont une activité sexuelle maximale. Bigna et al. [2] également rapportent une prévalence plus élevée que la nôtre; 10,2% chez les femmes enceintes dans un milieu rural à l’Extrême Nord du Cameroun. Ceci pourrait se justifier par leur niveau d’instruction bas, la méconnaissance des modes de transmission de l’hépatite B, la rareté des centres de dépistage et que la plupart d’entre elles étaient issues de ménages polygamiques. Le *Chlamydia trachomatis* est l’infection bactérienne sexuellement transmissible la plus répandue dans le monde. La distribution de cette infection a été de 22,52% dans notre étude. Une autre étude réalisée au Sénégal par Stum-Ramirez et al [9] sur les prostituées rapporte une prévalence de 28,5 %. Nos résultats sont proches de ceux rapportés dans ce pays, bien que dans cette dernière étude les auteurs ont utilisé une technique plus sensible que la nôtre (la polymerase chain reaction) sur des prélèvements endocervicaux ce qui a permis la détection des infections précoces. Dans l’infection à Chlamydia la réponse immunitaire est spécifique au sérotype, ce qui permet des réinfections avec d’autres sérotype ou avec le même sérotype ayant subit une mutation antigénique [10]. Même les patients antérieurement traités de chlamydia sont à risque de se réinfecter d’après Stevens et al. [11]. Le taux de réinfection dans notre étude était de 4,94%. Jolandes et al [10] rapportent un taux de 30 à 50% de l’infection à *Chlamydia trachomatis* chronique suite à une infection persistante ou à une réinfection chez des jeunes adolescents. Ce taux très élevé par rapport au nôtre peut se justifier par le jeûne âge de ces adolescents qui le plus souvent entretiennent des rapports sexuels non protégés et changent fréquemment de partenaires sexuels. La distribution de l’infection syphilitique était de 0,54% dans notre étude, (1/182). Nos résultats sont similaires à ceux de Carles et al. [12] en Guyanne française avec une prévalence de 0,5% et sont moins élevés que ceux trouvés par Bouba et al. [6] chez les femmes au Nord du Cameroun avec une prévalence de 5,5%. Ce taux élevé rapporté dans cette dernière étude pourrait se justifier par le fait que les femmes de cette région du Nord sont pour la plupart sous scolarisées et issues de ménages polygames ce qui augmenterait le risque potentiel de transmission de cette affection.

Dans une étude, Mawak et al. [13] ont trouvé, une association significative entre le *Chlamydia trachomatis* et les autres infections sexuellement transmissibles avec un taux de 41,46%. Dans notre étude, le taux de coïnfection *Chlamydia trachomatis* et hépatite virale B était de 2,74%. Rares ont été les études qui ont rapporté cette coïnfection au Cameroun. Néanmoins, le taux de coinfection que nous avons trouvé n’est pas négligeable. Il nous rappelle que les personnes infectées par le *Chlamydia trachomatis* doivent être aussi dépistées sur les autres infections sexuellement transmissibles en particulier l’hépatite B. S’agissant des modes de transmission de ces affections 67,57% et 70,87% de femmes ne connaissent pas la voie de transmission sexuelle pour le *Chlamydia trachomatis* et pour l’hépatite virale B respectivement, tandis que 91,2 % des femmes connaissent la transmission sexuelle pour la syphilis. Cette dernière est la seule infection où les femmes éduquées (69,23%) connaissent mieux la transmission sexuelle contre 25,27% et 30,21% respectivement pour l’hépatite B et le *Chlamydia trachomatis*. Ceci pourrait s’expliquer par le fait que la syphilis était connue de l’humanité depuis le XV^ème^ siècle [14], alors que les deux autres affections sont de découverte récente.

## Conclusion

L’hépatite B et le *Chlamydia trachomatis* constituent un problème de santé publique au Cameroun. La coïnfection *Chlamydia trachomatis* hépatite virale B n’est pas à minimiser. La découverte de l’infection à *Chlamydia trachomatis* chez une patiente doit susciter le dépistage de l’hépatite virale B.

### Etat des connaissances actuelles sur le sujet

- Les infections sexuellement transmissibles sévissent dans les pays en voie de développement;
- L'infection à *Chlamydia trachomatis* est l’infection sexuellement; transmissible d’origine bactérienne la plus répandue dans le monde;
- La syphilis est en recrudescence de par le monde.

### Contribution de notre étude à la connaissance

- Etude de la coïnfection *Chlamydia trachomatis*/hépatite virale B au Cameroun;
- Prévalence de la réinfection au *Chlamydia trachomatis* chez la femme Camerounaise;
- Connaissance de la femme camerounaise sur la transmission sexuelle de l’hépatite virale B, le *Chlamydia trachomatis*, la syphilis.
